# The Role of the Rad55–Rad57 Complex in DNA Repair

**DOI:** 10.3390/genes12091390

**Published:** 2021-09-08

**Authors:** Upasana Roy, Eric C. Greene

**Affiliations:** Department of Biochemistry & Molecular Biophysics, Columbia University, New York, NY 10032, USA; ur2129@cumc.columbia.edu

**Keywords:** homologous recombination, Rad51, Rad51 paralogs, Rad55–Rad57, recombination mediators

## Abstract

Homologous recombination (HR) is a mechanism conserved from bacteria to humans essential for the accurate repair of DNA double-stranded breaks, and maintenance of genome integrity. In eukaryotes, the key DNA transactions in HR are catalyzed by the Rad51 recombinase, assisted by a host of regulatory factors including mediators such as Rad52 and Rad51 paralogs. Rad51 paralogs play a crucial role in regulating proper levels of HR, and mutations in the human counterparts have been associated with diseases such as cancer and Fanconi Anemia. In this review, we focus on the *Saccharomyces cerevisiae* Rad51 paralog complex Rad55–Rad57, which has served as a model for understanding the conserved role of Rad51 paralogs in higher eukaryotes. Here, we discuss the results from early genetic studies, biochemical assays, and new single-molecule observations that have together contributed to our current understanding of the molecular role of Rad55–Rad57 in HR.

## 1. Introduction

DNA double-stranded breaks (DSBs) are highly toxic DNA lesions that, if misrepaired, can lead to gross chromosomal rearrangements and genome instability. Accurate repair of DSBs requires homologous recombination (HR), which uses a homologous DNA sequence elsewhere in the genome as a template to restore genetic information around the DNA break site [1,2,3]. Given its importance in maintaining genomic stability, HR has been conserved from bacteria to humans, and a deficiency of HR is associated with diseases such as Fanconi Anemia and multiple types of cancer [4,5].

**Figure 1:**
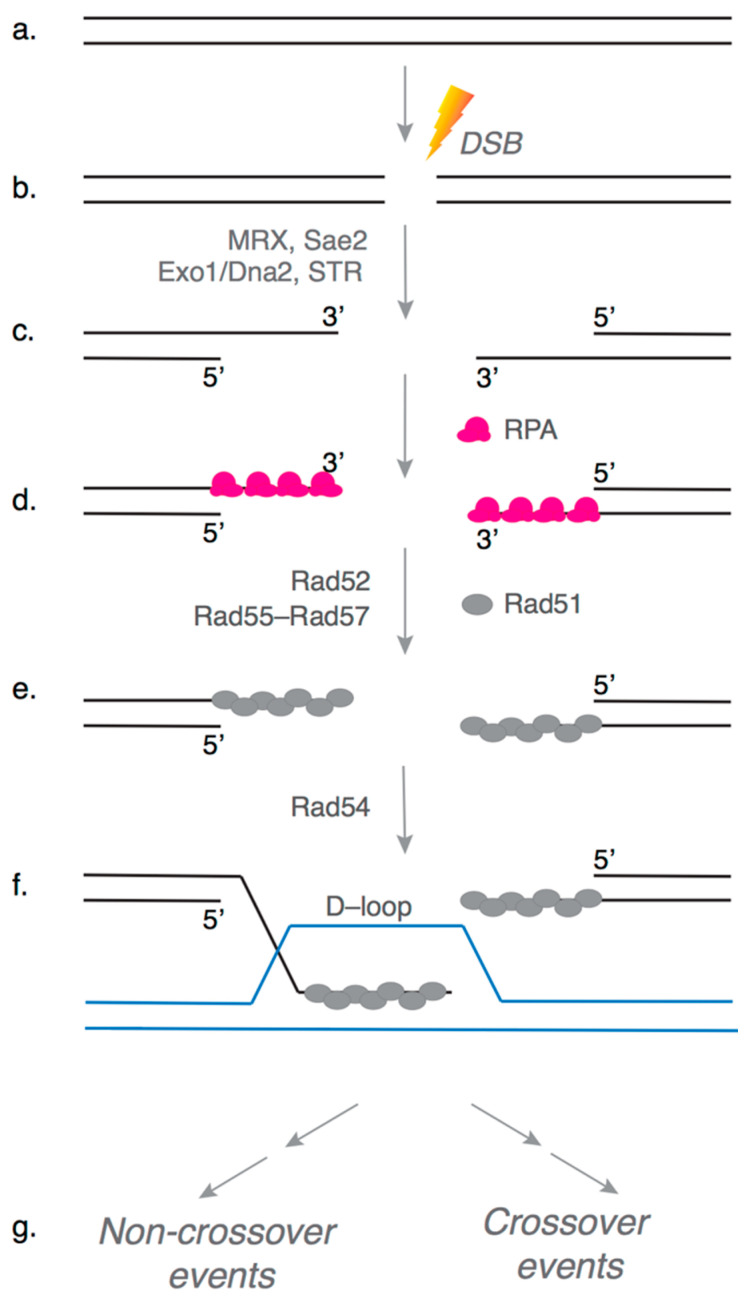
Early steps of Homologous Recombination (HR). A simplified schematic of the early steps of the HR pathway is shown here. (**a**,**b**) Genomic DNA can be damaged by a variety of agents to generate a double-stranded break (DSB). (**c**) Multiple nucleases resect the DSB ends, generating 3′ single-stranded DNA (ssDNA) overhangs, such as Mre11–Rad50–Xrs2 (MRX), Sae2, Exo1, Dna2 and Sgs1–Top3–Rmi1 (STR). (**d**) The overhangs are rapidly bound by the ssDNA binding protein RPA, and (**e**) Rad51 recombinase displaces RPA assisted by mediator proteins Rad52 and Rad55–Rad57 to form the presynaptic complex. (**e**) The presynaptic complex searches the genome for a homologous DNA template with the help of DNA translocase Rad54. (**f**) Rad51 catalyzes DNA strand invasion to form a D-loop intermediate with the homologous template. (**g**) The broken DNA end within the D-loop is extended by DNA polymerases, and the resulting repair intermediate can be resolved via one of several mechanistically distinct pathways (not shown) to produce crossover or non-crossover events.

The key steps of HR are conserved across eukaryotes, and here we briefly describe the early steps of HR in *S. cerevisiae* (Figure 1). First, DNA around the break site is processed by nucleases and helicases in two stages to expose long 3′ single-stranded DNA (ssDNA) overhangs. The initial short-range resection is carried out by Mre11–Rad50–Xrs2 (MRX) along with Sae2, followed by long-range resection by Exo1 and/or Dna2 with Sgs1–Top3–Rmi1 (STR) [6] (Figure 1b,c). The 3′ ssDNA overhangs are rapidly bound by replication protein A (RPA), which is a highly abundant protein with a high affinity for ssDNA (Figure 1d). The Rad51 recombinase, the key catalytic protein in HR, then displaces RPA from ssDNA with the help of mediator proteins such as Rad52 and Rad55–Rad57, to form a long helical nucleoprotein filament called the presynaptic filament (Figure 1e). Rad51 searches the genome for a homologous target, by sampling the sequence complementarity of the underlying ssDNA in the presynaptic filament with genomic DNA sequences, in a process catalyzed by the DNA translocase Rad54 [7]. Once a homologous DNA target is found, Rad51 catalyzes strand invasion to form a stable D-loop intermediate where the broken 3′ DNA end is paired with an intact homologous template to allow extension by DNA synthesis (Figure 1f). The broken 3′ DNA end is extended by DNA polymerases using the homologous DNA as a template to restore the DNA sequence around the break site. The extended D-loop intermediate can then be processed by one of several potential pathways to produce crossover or non-crossover products [1,2] (Figure 1g).

### 1.1. Mediator Proteins in HR

RPA is an important factor participating in the DNA transactions that take place during HR, with seemingly contradictory effects on HR. In the initial stages of DNA resection, RPA stimulates formation of 3′ ssDNA overhangs, and rapidly binds the newly exposed ssDNA, protecting the ends from further degradation by nucleases and removing any secondary structure. The RPA–ssDNA filament also plays an important role in checkpoint signaling by recruiting Mec1–Ddc2 to activate the DNA damage checkpoint [8]. In addition, RPA facilitates later steps of HR such as D-loop formation, as revealed by in vitro strand exchange assays. Strand exchange products form efficiently when RPA is added along with the homologous dsDNA duplex to pre-assembled Rad51–ssDNA filaments, whereas omission of RPA at this step leads to a marked reduction in product formation [9,10]. The ability of RPA to resolve secondary structures in the underlying ssDNA also appears to be important for the next steps of HR. Rad51–ssDNA filaments assembled in the absence of RPA are less contiguous, as observed in electron micrographs, suggesting that removal of ssDNA secondary structures by RPA facilitates Rad51 polymerization [11].

Paradoxically, however, pre-binding of ssDNA by RPA strongly inhibits the binding of Rad51 to ssDNA—a necessary next step for homologous recombination. In vitro reactions where RPA is added before, or along with Rad51 to ssDNA, show a dramatic reduction in the formation of Rad51–ssDNA filaments and strand exchange products [9,12,13]. To overcome this inhibitory effect of RPA, a class of HR regulators called mediators are required to facilitate Rad51 assembly onto RPA-coated ssDNA [2,3,14]. In *S. cerevisiae*, Rad52 and Rad51 paralogs act as mediators and are crucial for HR in vivo, as deletion of *RAD52* or *RAD51* paralogs sensitizes cells to DNA damaging agents, and impairs formation of ionizing radiation (IR)-induced Rad51 foci [3,14]. Rad52 is considered a classical mediator of HR and it promotes loading of Rad51 onto RPA–ssDNA through interactions with both Rad51 and RPA [12,15,16]. The Rad51 paralogs in budding yeast include Rad55 and Rad57, which function as a heterodimer, and Shu1, Csm2, and Psy3, which form a heterotetramer along with Shu2 called the SHU complex [14].

The role of recombination mediators is highly conserved and essential for maintaining proper levels of HR in vivo. In nematodes, BRC-2 and the RAD-51 paralog complex RFS-1/RIP-1 act as mediators of recombination and promote formation of the RAD-51 filament [14,17]. In mammalian cells, BRCA-2 acts as the primary recombination mediator, while RAD52 appears to play only a secondary role [1,2,17]. In addition, five canonical Rad51 paralogs mediate HR—RAD51B, RAD51C, RAD51D, XRCC2, and XRCC3—which function in several subcomplexes: BCDX2 (RAD51B–RAD51C–RAD51D–XRCC2), CX3 (RAD51C–XRCC3), and RAD51C–RAD51–BRCA2–PALB2 [1,2,17]. The importance of their function is highlighted by the link between mutations in RAD51 paralogs and cancer-related diseases. Mutations in RAD51C and XRCC2 are associated with Fanconi Anemia, which causes bone marrow failure and a predisposition to leukemia and solid tumors, and mutations in all RAD51 paralogs have been associated with multiple types of cancer including mostly breast and ovarian cancers [4,14,17]. However, the mechanistic basis for the mediator activity of Rad51 paralogs is not well defined, and continues to be an active area of research.

### 1.2. Rad51 Paralogs

The central recombinase in HR is conserved across all three domains of life—RecA in bacteria, and the *RAD51* superfamily in archaea/eukaryotes. Unlike bacteria, both archaea and eukaryotes have a diversified set of Rad51-like proteins that resulted from early gene duplication events [18]. Based on phylogenetic analysis, RecA/Rad51-like proteins can be divided into three subfamilies: (i) RecA, found in bacteria and some eukaryotic organelles; (ii) RADα, found in archaea and eukaryotes that display a highly conserved recombinase function; and (iii) RADβ, also found in archaea and eukaryotes, but with highly divergent functions [18]. However, all RecA/Rad51-like proteins share a conserved central core domain of ~230 amino acids. Within this RecA/Rad51 core domain lie two highly conserved Walker A and Walker B motifs responsible for coupling ATP binding and hydrolysis to DNA binding activities [18,19].

The eukaryotic mitotic recombinase Rad51 and meiotic recombinase Dmc1 belong to the RADα subfamily, whereas the RADβ subfamily consists of the *RAD51-*related genes with highly divergent N– and C–termini called Rad51 paralogs. Unlike Rad51 and Dmc1, the Rad51 paralogs are not DNA recombinases, even though they share homology with the RecA/Rad51 ATPase core domain. However, Rad51 paralogs facilitate Rad51-dependent HR, and are important for the formation of damage-induced Rad51 foci and resistance to DNA-damaging agents [17]. Deletion of Rad51 paralogs in yeast sensitizes cells to DNA-damaging agents, deletion of vertebrate paralogs results in embryonic lethality, and mutations in the human paralogs are associated with multiple types of cancer [14]. In budding yeast, five Rad51 paralogs have been identified: Rad55, Rad57, Shu1, Csm2 and Psy3. These Rad51 paralogs function in two distinct complexes—the Rad55–Rad57 heterodimer, and the SHU complex comprising of Shu1, Shu2, Csm2 and Psy3. The two paralog complexes promote HR, but their precise molecular functions are not fully understood yet. Rad55–Rad57 is considered the canonical Rad51 paralog complex, and serves as an important model for understanding the role of eukaryotic Rad51 paralogs. Here, we will review the early observations and significant advances made in our understanding of the role of Rad55–Rad57 in mitotic DSB repair.

## 2. Rad55–Rad57: Early Clues to Their Function in HR

Rad55 and Rad57 were first identified in a genetic screen designed to isolate mutants that conferred sensitivity to X-rays [20]. Sequencing of the *RAD55* and *RAD57* genes revealed a similarity to *RAD51*, primarily in the conserved RecA/Rad51 core domain containing the Walker A and B motifs [21,22]. Rad55 and Rad57 share a 20–30% amino acid sequence identity with Rad51, mostly limited to the ATPase core domain [4,14].

Early genetic studies suggested that Rad55 and Rad57 may function together in a complex. Mutations in either *RAD57* or *RAD55* conferred similar sensitivities to IR, and the *rad55 rad57* double mutant behaved similarly to either single mutant [23,24]. Strong interactions between the Rad55 and Rad57 proteins were confirmed by yeast two-hybrid analysis, which also revealed interactions between Rad55 and Rad51, but not between Rad57 and Rad51 [23,24]. A direct physical association between Rad55 and Rad57 was validated by the first biochemical purification and characterization of the Rad55–Rad57 complex, which demonstrated that Rad55–Rad57 forms a highly stable heterodimer with an equilibrium dissociation constant <0.2 nM [9].

Another set of early observations indicated a key difference between Rad51 and its paralogs, namely, Rad55 and Rad57 do not form extended filaments on DNA, and they cannot catalyze strand exchange. Unlike Rad51, neither Rad55 nor Rad57 display evidence of any self-interaction in a yeast two-hybrid assay, indicating that they do not polymerize [23,24]. Due to its filament-forming ability, expression of the Rad51 ATPase mutant *rad51-K191A* in an otherwise wild-type strain background displays a dominant negative phenotype, and sensitizes cells to IR [25]. A similar expression of the Rad55 ATPase mutant *rad55-K49A* does not show a dominant negative phenotype, supporting the idea that the Rad55–Rad57 complex does not form filaments [24]. A systematic biochemical analysis of the Rad55–Rad57 complex also established that, despite sharing homology with the Rad51 core domain, the complex does not have recombinase activity and does not catalyze strand exchange in vitro [9]. These observations set up the early framework for investigating the role of Rad55–Rad57 in HR.

## 3. Cellular Defects in *rad55 rad57* Mutants

Genetic studies have provided a wealth of information about the function of Rad55–Rad57. *rad55* and *rad57* mutants have broadly similar phenotypes, showing an increased sensitivity to DNA damaging agents such as IR and methyl methanesulfonate (MMS), but not UV irradiation, as measured by quantitative cell survival assays [20,23]. Mutants of *RAD55* and *RAD57* show a reduced rate of gene conversion in response to a HO-inducible DSB, and reduced rates of IR-induced recombination [23,26]. *rad55* and *rad57* mutants are also defective in mating-type switching and sporulation, showing a strong defect in meiotic viability [24,26]. Together, these observations have confirmed the role of Rad55 and Rad57 as important factors in mitotic and meiotic recombination.

The study of recombination foci has been valuable in understanding the choreography of HR proteins at DSB sites [27]. In response to IR-induced DSBs, *rad55Δ* cells show no defect in Rad52–CFP foci formation, but form YFP–Rad51 foci that are less bright than those observed by fluorescence microscopy in wild-type cells [28,29]. Using a YFP-tagged version of the *RAD55 RAD57* suppression mutant *rad51–I345T*, Fung et al. found that *rad57Δ* cells show a marked reduction in the number of YFP–Rad51 foci in response to the DNA-damaging agent camptothecin [30]. Mutations in *RAD55* and *RAD57* also lead to a loss of Rad51 foci formation in meiotic cells [31]. Experiments with fluorescently tagged versions of Rad55 in *rad51Δ* cells show that Rad55 foci formation requires Rad51 [28]. To understand which particular stage of HR requires Rad55–Rad57 activity, Sugawara et al. induced a single unrepairable DSB (lacking a donor template) using a GAL-inducible HO endonuclease, and monitored the kinetics of Rad51 recruitment around the DSB site using chromatin immunoprecipitation [32]. In cells depleted of Rad55, recruitment of Rad51 at the break site was significantly diminished and delayed [32]. Together, these studies established that Rad55 is recruited to DSB repair sites in a Rad51-dependent manner, and is required for the formation and stabilization of Rad51 recombination foci.

While most genetic assays show identical phenotypes for *rad55* and *rad57* mutants, mutations in their putative ATP binding sites reveal a distinction between the two paralogs. *rad55–K49R* cells, which contain a mutation in their ATPase active site that should block ATP hydrolysis, are more sensitive to IR in quantitative cell survival assays than wild-type cells, but more resistant than *rad55Δ* mutants. In contrast, an equivalent mutation in the Rad57 ATPase domain does not sensitize cells to IR, as *rad57–K131R* cells show similar resistance to IR as wild-type cells [24]. This suggests that the ATPase activity of Rad55 is important in the function of the Rad55–Rad57 complex, whereas ATPase activity of Rad57 is dispensable. New single-molecule studies have now shown that ATP hydrolysis by the complex is required for its dissociation from the Rad51–ssDNA filaments (see below) [33].

### 3.1. Suppression of Defects in RAD55 RAD57 Mutants

Multiple factors suppress the defects of *rad55 rad57* mutants to various levels, providing valuable information regarding the underlying deficiencies in these cells. The IR sensitivity of *rad55* and *rad57* mutants can be strongly suppressed by overexpression of Rad51, and to a lesser extent by overexpression of Rad52 [23,24,26]. Expression of *rad51–I345T*, a gain-of-function mutant with higher affinity for DNA, also partially suppresses the IR sensitivity of *rad57* mutants as well as their defect in mating-type switching [34]. Expression of both mating-type alleles in haploid or diploid cells also suppresses the IR sensitivity and interhomolog recombination defect of *rad55 rad57* mutants; however, the mechanistic basis for this remains poorly understood [26,35].

Deletion of *SRS2*, the 3′→5′ helicase with potent anti-recombinase activity [36,37,38], also partially suppresses the IR sensitivity of *rad55Δ* and *rad57Δ* cells [13,30]. Srs2 is a classical anti-recombinase that disrupts Rad51–ssDNA filaments as it translocates on ssDNA, by stimulating the rapid dissociation of Rad51 filaments [36,37,38,39]. This has led to a model where Rad55–Rad57 is thought to have two functions in HR: first, as a mediator that relieves the inhibition imposed by RPA on Rad51 filament assembly, and second, as protecting Rad51–ssDNA filaments from disruption by Srs2.

To address whether the primary role of Rad55–Rad57 is limited to early HR during Rad51 filament assembly, or whether the paralog complex has additional roles in the later steps of HR, Fung et al. asked whether combining factors that promote Rad51 filament formation could completely suppress the defects in *rad55Δ* and *rad57Δ* cells [30]. A quantitative assay measuring DSB-induced gene conversion shows a severe defect in *rad57* mutants compared to wild-type, which can be partially suppressed by expression of *rad51–I345T* or *SRS2* deletion or MAT heterozygosity. A combination of three or more such factors leads to almost complete suppression of the IR defects in *rad55Δ* and *rad57Δ* cells, and near-wild-type levels of DSB-induced gene conversion rates, resistance to IR and resistance to the genotoxic agent camptothecin [30]. Due to the near-complete suppression of *rad55* and *rad57* defects seen by combining factors that promote Rad51 filament assembly, it is believed that the main role of Rad55 and Rad57 is at the level of Rad51 filament formation and maintenance rather than the later steps in HR.

### 3.2. Cold-Sensitive Phenotypes in RAD55 and RAD57 Mutants

One of the earliest observations in *rad55* and *rad57* mutants set them apart from all other genes in the *RAD52* epistasis group—many of the defects seen in *rad55* and *rad57* mutants were exacerbated at lower temperatures. *RAD55* and *RAD57* null mutants are sensitive to X-rays, and their sensitivity increases at lower temperatures [26]. At 30 °C, *rad57* mutants are 10- to 100-fold more resistant to γ rays compared to *rad51* mutants. However, at 20 °C, *rad57* mutants are as sensitive to γ rays as *rad51* mutants, and the *rad51 rad57* double mutants are as sensitive as the single mutants [24,34]. Although temperature-dependent sensitivity has been observed in other mutant proteins, the underlying basis is not well defined. Cold-sensitivity is a property often associated with proteins that stabilize large multiprotein complexes [40,41]. It was therefore suggested that the cold-sensitive phenotype seen in *rad55 rad57* mutants is due to their role in stabilizing a multiprotein or a protein–DNA recombination intermediate that is otherwise unstable at low temperatures [20,23,24,26].

### 3.3. Role of Rad55–Rad57 in Spontaneous Recombination

In addition to the repair of DSBs induced by DNA-damaging agents such as IR, Rad55–Rad57 also plays an important role in spontaneous recombination, which consists of repair events occurring in the absence of exogenous DNA-damaging agents. These events likely represent the repair of replication-associated damage such as long ssDNA gaps, and stalled or collapsed replication fork structures. *rad57* mutants show a severe defect in an assay measuring spontaneous gene conversion between direct repeats, and in contrast to their defect in IR-induced recombination, the spontaneous recombination defect in *rad57* mutants is not suppressed by Rad51 overexpression, *SRS2* deletion or MAT heterozygosity [30,35]. In addition, the spontaneous recombination defect in *rad57* mutants is not a cold-sensitive phenotype, unlike the defect in the IR-induced rate of recombination, which can be suppressed by higher temperatures [35]. Postreplication Repair Pathway (PRR) is an alternative mechanism for the repair of stalled forks that relies on DNA synthesis by the highly mutagenic translesion synthesis (TLS) polymerases. Apart from lower rates of spontaneous recombination, *rad57* mutants also display an increased number of spontaneous mutations that are dependent on the TLS polymerase REV3 [42]. These observations are consistent with the idea that HR and PRR compete for the repair of ssDNA gaps, and indicate that Rad55–Rad57 plays an important role in promoting their accurate repair via HR.

Since the DSB-induced recombination defect in *rad55 rad57* cells can be rescued by factors that promote Rad51 activity, it is believed that the predominant role of Rad55–Rad57 in DSB repair is its mediator activity. This appears to be in contrast with the role of Rad55–Rad57 in spontaneous recombination, which is not cold-sensitive and not strongly suppressed Rad51 overexpression or *SRS2* deletion [30,35]. These observations present two possible scenarios: Rad55–Rad57 may have a unique role in spontaneous recombination that is distinct from its function in DSB repair, and/or there is a greater dependence on the mediator activity of Rad55–Rad57 during spontaneous recombination compared to damage-induced DSB repair [35].

## 4. Regulation of Rad55–Rad57

DNA damage checkpoints are essential for the coordination of DNA repair and cell cycle progression, and therefore essential for the maintenance of genomic integrity. Multiple protein sensors monitor the genome for DNA damage and use a network of kinase cascades to impose a transient cell cycle arrest that allows timely and proper repair to occur. Among this complex network of DNA damage sensing and signaling proteins is the key regulator Mec1 kinase, which is the yeast homolog of mammalian ATM kinase [43]. Mec1 activates the effector kinase Rad53, which in turn regulates a wide spectrum of pathways in response to DNA damage [44]. Rad55 is strongly phosphorylated in a Mec1-dependent manner in response to MMS, IR, and to a lesser extent, in response to HU treatment, as observed by electrophoretic mobility shift assays (EMSA) [45]. A majority of the sites in Rad55 are phosphorylated in a Rad53-dependent manner, and at least one site is directly phosphorylated by Mec1 independent of Rad53 [45,46,47]. Some of the phosphorylation sites in Rad55 that have been identified include: serines 2, 8, 14, 19, and 20 in the N–terminus, and serine 378 in the C–terminus, which are all phosphorylated in response to MMS. On the other hand, the C–terminus site serine 404 is phosphorylated constitutively, in the presence or absence of MMS [48]. The effect of the phosphorylation varies as Rad55 phosphorylation at serines 2, 8, and 14 appears to affect protein function but not stability, whereas phosphorylation of serines 19, 20, and 378 affects protein stability [47,48]. In cells undergoing S/G2 phase, or cells exposed to MMS, Rad53 gets activated by Mec1 and phosphorylates Rad55–serine 2, 8, and 14, which affects Rad55 activity [49]. A Rad55 triple mutant in which serines 2, 8, and 14 are mutated to alanine shows no growth defect in the absence of DNA damage, but in response to chronic MMS exposure shows increased sensitivity and a slow growth phenotype compared to wild-type cells, although it is more resistant than *rad55* null mutants [49]. Cells expressing Rad55–S2A, S8A, and S14A show a strong defect in the recovery of replication forks stalled due to MMS exposure, as measured by completion of DNA synthesis in pulsed-field gel electrophoresis and flow cytometry assays [49]. These results suggest that the phosphorylation of the three N–terminal resides is important for optimal Rad55 activity in DNA repair and resistance to genotoxic agents.

A single DSB in G1-arrested cells is also sufficient to activate Mec1 kinase, but not the Rad53-dependent arm of checkpoint signaling. Under these conditions, Mec1 directly phosphorylates Rad55 at serine 378 without activation of Rad53 [46]; however, the functional consequence of serine 378 phosphorylation is unclear and remains to be determined. Assigning a clear mechanistic function to these post-translational modifications has been challenging due to the numerous modification sites, low cellular abundance of Rad55, and the subtle effects observed in genetic assays. Mapping of additional phosphorylation sites and determining their functional consequence will be valuable for understanding how Rad55–Rad57 activity is regulated in response to DNA damage.

Relatively little is known about the transcriptional regulation of Rad55 and Rad57. In a high-throughput genetic interaction screen, *RAD55* phosphorylation site mutants were used to identify the role of mRNA nonsense-mediated decay (NMD) in post-transcriptional regulation of Rad55–Rad57 levels [48]. In wild-type cells, NMD suppresses HR by degrading Rad55 and Rad57 mRNA, and the steady-state levels of these transcripts are regulated by NMD both in the presence and absence of DNA damage. Disruption of the NMD pathway suppresses the MMS sensitivity of a Rad55 mutant in which serines 2, 4, 8, 19, and 20 are mutated to alanine. Deletion of NMD factors increases the half-life of the Rad55 mRNA transcript, and leads to hyperrecombination in the absence of external DNA damage [48]. However, whether Rad55 and Rad57 transcript and protein levels are specifically regulated in response to DNA damage remains to validated.

## 5. Molecular Role of Rad55–Rad57 in HR

Purification of the Rad55–Rad57 complex has been a challenge due to its low cellular abundance and poor solubility. Therefore, only a few biochemical studies have informed our current understanding of the molecular function of this paralog complex. Cellular abundance of Rad55–Rad57 is ~10% that of Rad51 levels, and although Rad55 and Rad57 exist as a highly stable heterodimer, they are not stably associated with Rad51 in yeast cell lysates [9]. In vitro strand exchange assays show that Rad55–Rad57 cannot catalyze strand exchange, despite sharing homology with the RecA/Rad51 recombinase core domain. However, these assays provided the first biochemical evidence that Rad55–Rad57 acts as a mediator, and relieves the inhibition of RPA on Rad51-mediated strand exchange [9]. The mechanism does not involve a direct stimulation of Rad51 activity, since reactions without RPA are not affected by the addition of Rad55–Rad57 [9,12]. Rad55–Rad57 exhibits only a weak ATPase activity, which, in contrast to the ATPase activity of Rad51, is not stimulated by DNA cofactors [9,13]. Observations from in vitro DNA binding assays suggest that Rad55–Rad57 may increase the stability of Rad51–ssDNA filaments. Assembly of Rad51–ssDNA filaments with a sub-saturating ratio of Rad51 to ssDNA produces filaments that are highly salt-labile, but including sub-stoichiometric amounts of Rad55–Rad57 stabilizes the Rad51–ssDNA complexes even under high-salt conditions [13]. This stabilization effect may be due to the formation of longer continuous filaments, or due to remodeling of the Rad51–ssDNA filament structure, similar to that observed with the *C. elegans* paralogs RFS-1/RIP-1 [50].

Another specific stabilizing role ascribed to Rad55–Rad57 is as an antagonist of the antirecombinase Srs2, which disrupts Rad51–ssDNA filaments as it translocates in a 3′→5′ direction on ssDNA [13,36,37,38]. In genetic assays, sensitivity of *rad55* and *rad57* deletion mutants to IR and MMS is partially suppressed by deletion of *SRS2*, and in vitro pull-down experiments show that Rad55–Rad57 physically interacts with Srs2 [13,29]. Pull-down experiments using ssDNA conjugated to magnetic beads show that the fraction of Rad51 that remains bound to ssDNA is greatly reduced in the presence of Srs2. In contrast, inclusion of Rad55–Rad57 leads to an increase in the fraction of Rad51 that remains bound to ssDNA, even in the presence of Srs2 [13]. However, Rad55–Rad57 does not inhibit Srs2 ATPase activity or helicase activity directly, as the suppression of Srs2 activity depends on the presence of Rad51 [13]. The model proposed to account for these observations suggests that Rad55–Rad57 is incorporated into a growing Rad51–ssDNA filament and acts as a barrier to Srs2 translocation, thereby protecting the filaments against disruption by Srs2 [13].

The Rad55–Rad57 complex also plays an important role in mediating interactions of Rad51 with other positive regulators of HR such as the SHU complex, which is comprised of Shu2 and the Rad51 paralogs Shu1, Csm2, Psy3 [51]. Yeast two-hybrid assays and in vitro pull-down experiments show that Rad55 interacts with Csm2, and this interaction is required for the association of the SHU complex with Rad51, as well as Rad52 [12,51]. The interaction of Csm2 with Rad55 is functionally significant, as demonstrated by studies with the Csm2–F46A mutant, which maintains its interactions within the SHU complex and with DNA, but does not interact with Rad55 [12]. While a wild-type SHU complex stimulates Rad51 assembly onto RPA–ssDNA in the presence of Rad55, a complex containing the Csm2–F46A mutant that does not interact with Rad55 is unable to stimulate Rad51–ssDNA filament assembly [12]. Surprisingly, the Csm2–F46A mutant behaves similarly to the *csm2* null mutant in many respects: sensitivity to MMS, levels of Rad51-mediated gene conversion, and rates of mutation in a canavanine assay [12]. Therefore, Rad55 interaction with Csm2 appears to be indispensable for the cellular function of the SHU complex in HR.

## 6. Single-Molecule Perspective on Rad55–Rad57 Function

**Figure 2:**
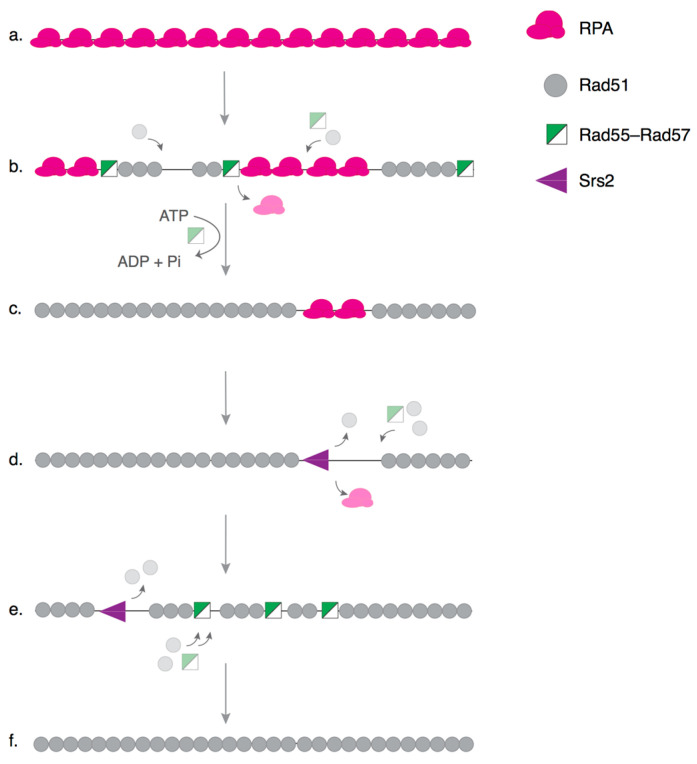
Model for the role of Rad55–Rad57 in presynaptic complex assembly. (**a**–**c**) Rad55–Rad57 stimulates Rad51 filament assembly onto RPA-coated single-stranded DNA (ssDNA) through transient binding interactions, and rapidly dissociates when Rad55 hydrolyzes ATP. (**d**) The antirecombinase Srs2 can disrupt Rad51 filaments as it translocates along ssDNA. (**e**,**f**) The Rad51–ssDNA filament is restored by the continuous stimulatory action of Rad55–Rad57, which acts as a molecular chaperone and leads to more extensive Rad51–ssDNA filament formation.

Single-molecule assays provide a powerful way to complement genetic and ensemble biochemical studies, as they can reveal a detailed picture of how individual reaction intermediates are regulated. Single-molecule studies of HR have provided a new level of mechanistic detail on how HR intermediates are generated and resolved [52,53,54]. We have used a single-molecule technique called DNA curtains to investigate the role of Rad55–Rad57 in Rad51–ssDNA filament dynamics [33]. DNA curtain assays are well suited for this purpose, as they allow direct imaging of Rad51–ssDNA filament dynamics in real time, using total internal reflection microscopy (TIRFM) [55,56,57,58]. Using this technique, we observed a chaperone-like behavior of the Rad55–Rad57 complex during Rad51 presynaptic filament assembly [33]. Rad55–Rad57 does not bind RPA–ssDNA or pre-assembled Rad51–ssDNA filaments, and instead rapidly co-assembles with Rad51 onto RPA–ssDNA filaments. However, the paralog complex does not remain stably bound to the Rad51–ssDNA filament, exhibiting only a transient interaction and dissociating rapidly. Rad55–Rad57 strongly stimulates the rate of Rad51 assembly, as well as the extent of RPA displacement by Rad51. This highly dynamic behavior of Rad55–Rad57 is also observed in vivo using FRAP experiments. The half-life of the paralog complex in IR-induced repair foci is ~3.2 s, whereas, under similar conditions, the half-life of Rad51 is ~87 s [59]. Dissociation of the Rad55–Rad57 complex from the Rad51–ssDNA filaments requires ATP hydrolysis by Rad55, as the half-life of the Rad55-K49R complex is ~3 times slower than the wild-type complex, whereas the half-life of the Rad57-K131R complex is similar to that of the wild-type complex. Visualization of Srs2 activity on Rad51–ssDNA filaments in the presence of Rad55–Rad57 suggests that the paralog complex does not directly inhibit Srs2 motor activity, but rather antagonizes it by promoting a faster reassembly of Rad51 onto ssDNA after Srs2 translocation [33]. The single-molecule results support a model where Rad51–ssDNA undergoes multiple cycles of assembly and disassembly whose overall rate is determined by the balance of mediators and Srs2, thereby ensuring dynamic control over the next steps (Figure 2). Notably, the transient binding and ability to stimulate recombinase filament assembly has also been observed for the nematode Rad51 paralogs RFS–1/RIP–1, suggesting that this chaperone-like mechanism of action for Rad51 paralogs may be conserved across eukaryotes [60].

## 7. Open Questions and Future Directions

Since their identification nearly 50 years ago, considerable advances have been made in understanding the role of Rad55–Rad57, which has also served as a model for understanding the conserved function of paralogs in higher eukaryotes. However, certain challenges and questions remain. Currently, structural information is unavailable for Rad55–Rad57 alone, or in a complex with Rad51–ssDNA, but it is of considerable interest to the field. Determining how Rad55–Rad57 associates with the Rad51–ssDNA filament will provide valuable information about the residues involved in these interactions, which will in turn provide access to more detailed information regarding its mechanism of action. Rad55–Rad57 also interacts with other HR regulators and Rad51 paralogs such as the SHU complex, but the specific physiological context of these interactions is not well defined. A mutational analysis to disrupt specific interactions will be extremely important for understanding the precise roles of Rad55–Rad57 and their contribution to regulating HR. However, such studies may pose a challenge due to the seemingly transient nature of Rad55–Rad57 interactions with other proteins, and may require trapping of the complex with non-hydrolyzable ATP analogs [33]. Another intriguing question that has not been addressed is whether binding of Rad55–Rad57 remodels the Rad51–ssDNA filaments, an effect that has been demonstrated for the *C. elegans* Rad51 paralog complex RFS-1/RIP-1 [50]. Structural studies would also be critical for determining whether the function of Rad55–Rad57 includes remodeling of the Rad51–ssDNA filament into a more “active/functional” state.

Current evidence indicates a difference in the role or dependence on Rad55–Rad57 in the context of DSB-induced recombination versus spontaneous recombination. Factors that suppress IR sensitivity of *rad55 rad57* mutants, such as Rad51 overexpression, higher temperature and *SRS2* deletion, do not suppress the defects in spontaneous recombination in these mutants [13,30]. Replication fork-associated ssDNA gap repair is thought to be the source of spontaneous recombination; therefore, these observations suggest that Rad55–Rad57 may have different roles in repair of DSBs versus replication-associated damage. However, the mechanistic basis for these observed differences is not well understood, and remains an area of interest.

Another fundamental unanswered question is regarding the division of labor between Rad51 mediators in HR. In *S. cerevisiae*, both Rad52 and Rad55–Rad57 act as mediators based on their ability to promote Rad51-mediated strand exchange in the presence of RPA [9,12,15]. However, they play non-redundant roles, since overexpression of Rad55–Rad57 does not rescue a *RAD52* deletion, and overexpression of Rad52 only mildly suppresses IR sensitivity of *RAD55* and *RAD57* deletion mutants [23,24]. Determining why multiple mediators are required, and the unique role of Rad55–Rad57 as a mediator, will inform our understanding of the functions of Rad51 paralogs, and contribute to a detailed understanding of how HR is regulated in eukaryotic cells.

Mutations in the human RAD51 paralogs are associated with multiple types of cancer and diseases such as Fanconi Anemia [4,14,17]. Therefore, determining the mechanism of action for the human Rad51 paralog complexes RAD51C–XRCC3 and RAD51B–RAD51C–RAD51D–XRCC2 is of significant interest, with important implications for human health and disease. Whether the function and mechanism of Rad55–Rad57 is conserved across the human counterparts, and the how mutations affect their function and regulation of HR, are compelling areas of future studies.
